# Supplementary material for: Reliability of ultrasonographic measurement of muscle architecture of the gastrocnemius medialis and gastrocnemius lateralis
Source: PLoS One. 2021 Sep 29;16(9):e0258014. doi: 10.1371/journal.pone.0258014 (PMC8480904; doi:10.1371/journal.pone.0258014)
Supplement: S1 Table — IQR = interquartile range; ICC = intra-class correlation coefficient; CI = confidence interval; RLOA = ratio limits of agreement; mm = millimetres; ° = degrees. (DOCX) [file pone.0258014.s001.docx]

**S1 Table.** **Inter-rater reliability: pairwise comparisons between Investigators A, B and C for fascicle length, pennation angle and muscle thickness of the gastrocnemius medialis and gastrocnemius lateralis muscles.**

|  | Gastrocnemius Medialis (1081 images) | | | |  | Gastrocnemius Lateralis (1064 images) | | | |
| --- | --- | --- | --- | --- | --- | --- | --- | --- | --- |
|  | Median (IQR) | | Mean Bias (RLOA) | Cohens |  | Median (IQR) | | Mean Bias (RLOA) | Cohens |
| *Fascicle Length* | |  |  |  |  |  |  |  |  |
| A vs B | 54.3 mm (48.6 - 62.6) | 54.8 mm (49.3 - 63.2) | -1.0% (-11.6 - 10.9) | 0.17 |  | 67.4 mm (58.5 - 76.9) | 67.7 mm (59.0 - 77.8) | -0.2% (-15.2 - 17.4) | 0.01 |
| A vs C | 54.3 mm (48.6 - 62.6) | 54.7 mm (49.3 - 62.6) | -0.8% (-13.1 - 13.2) | 0.09 |  | 67.4 mm (58.5 - 76.9) | 68.3 mm (59.9 - 78.0) | -1.3% (-17.0 - 17.5) | 0.12 |
| B vs C | 54.8 mm (49.3 - 63.2) | 54.7 mm (49.3 - 62.6) | 0.2% (-12.1 - 14.3) | 0.05 |  | 67.7 mm (59.0 - 77.8) | 68.3 mm (59.9 - 78.0) | -1.0% (-17.2 - 18.2) | 0.10 |
| *Pennation Angle* | |  |  |  |  |  |  |  |  |
| A vs B | 19.2° (16.6 - 21.7) | 18.8° (16.2 - 21.3) | 2.1% (-8.7 - 14.1) | 0.36 |  | 12.4° (10.2 - 14.2) | 12.0° (10.1 - 13.8) | 2.2% (-12.1 - 18.7) | 0.29 |
| A vs C | 19.2° (16.6 - 21.7) | 19.1° (16.4 - 21.8) | 0.4% (-12.1 - 14.6) | 0.04 |  | 12.4° (10.2 - 14.2) | 12.1° (9.9 - 13.9) | 2.2% (-13.3 - 20.5) | 0.25 |
| B vs C | 18.8° (16.2 - 21.3) | 19.1° (16.4 - 21.8) | -1.7% (-13.5 - 11.8) | 0.26 |  | 12.0° (10.1 - 13.8) | 12.1° (9.9 - 13.9) | 0.0% (-15.6 - 18.5) | 0.01 |
| *Thickness* | |  |  |  |  |  |  |  |  |
| A vs B | 16.4 mm (14.6 - 18.7) | 16.4 mm (14.5 - 18.6) | 0.5% (-2.7 - 3.8) | 0.30 |  | 13.0 mm (11.0 - 14.9) | 12.9 mm (10.9 - 14.7) | 0.9% (-3.2 - 5.1) | 0.40 |
| A vs C | 16.4 mm (14.6 - 18.7) | 16.4 mm (14.6 - 18.6) | 0.2% (-2.9 - 3.3) | 0.10 |  | 13.0 mm (11.0 - 14.9) | 13.0 mm (11.0 - 14.8) | 0.3% (-3.7 - 4.5) | 0.16 |
| B vs C | 16.4 mm (14.5 - 18.6) | 16.4 mm (14.6 - 18.6) | -0.4% (-3.6 - 3.0) | 0.19 |  | 12.9 mm (10.9 - 14.7) | 13.0 mm (11.0 - 14.8) | -0.5% (-4.8 - 3.9) | 0.22 |

IQR = interquartile range; ICC = intra-class correlation coefficient; CI = confidence interval; RLOA = ratio limits of agreement; mm = millimetres; ° = degrees
